# Energy landscape scheme for an intuitive understanding of complex domain dynamics in ferroelectric thin films

**DOI:** 10.1038/srep11625

**Published:** 2015-07-01

**Authors:** Tae Heon Kim, Jong-Gul Yoon, Seung Hyub Baek, Woong-kyu Park, Sang Mo Yang, Seung Yup Jang, Taeyuun Min, Jin-Seok Chung, Chang-Beom Eom, Tae Won Noh

**Affiliations:** 1 Center for Correlated Electron Systems, Institute for Basic Science (IBS), Seoul 151-742, Republic of Korea; 2 Department of Physics & Astronomy, Seoul National University (SNU), Seoul 151-742, Republic of Korea; 3 Department of Physics, University of Suwon, Hwaseong, Gyeonggi-do 445-743, Republic of Korea; 4 Department of Materials Science and Engineering, University of Wisconsin-Madison, Madison, WI 53706, USA; 5 Department of Physics, Soongsil University, Seoul 156-743, Republic of Korea

## Abstract

Fundamental understanding of domain dynamics in ferroic materials has been a longstanding issue because of its relevance to many systems and to the design of nanoscale domain-wall devices. Despite many theoretical and experimental studies, a full understanding of domain dynamics still remains incomplete, partly due to complex interactions between domain-walls and disorder. We report domain-shape-preserving deterministic domain-wall motion, which directly confirms microscopic return point memory, by observing domain-wall breathing motion in ferroelectric BiFeO_3_ thin film using stroboscopic piezoresponse force microscopy. Spatial energy landscape that provides new insights into domain dynamics is also mapped based on the breathing motion of domain walls. The evolution of complex domain structure can be understood by the process of occupying the lowest available energy states of polarization in the energy landscape which is determined by defect-induced internal fields. Our result highlights a pathway for the novel design of ferroelectric domain-wall devices through the engineering of energy landscape using defect-induced internal fields such as flexoelectric fields.

Domain-wall (DW) motion in ferroic materials has been the subject of abundant researches^1^,^2^,^3^,^4^,^5^,^6^,^7^,^8^,^9^,^10^,^11^,^12^ and is critical to many device applications^4^,^5^,^6^ because it significantly affects dielectric, piezoelectric, ferroelectric, and magnetic properties^4^. Interestingly, the interaction of DWs with defects induces many intriguing phenomena, including complex but universal pinning-dependent field-driven DW dynamics^8^,^9^,^10^,^11^ and the wide spread occurrence of Rayleigh behavior in disordered ferroics^12^,^13^,^14^ by which the nonlinear responses of magnetic, piezoelectric and dielectric properties at low fields are analyzed. In addition, DW itself can have functional properties, such as electronic conductivity of ferroelectric DWs^15^, the magnetic moments of ferroelectric DWs in antiferromagnets^16^ and electric polarization of magnetic DWs^17^, that offer potential for active nanodevice applications^4^,^5^,^6^,^18^. Most of next-generation nanoscale non-volatile memory and logic devices based on DW motion rely on the controlled movement of DWs in ferroic systems^4^,^5^,^6^,^18^,^19^,^20^. In magnetic systems, a well controlled DW motion has been achieved by introducing artificial defects to engineer the energy landscape^21^. Such a deterministic DW motion actually has been a longstanding question related to microscopic return point memory, i.e., whether one point on the major hysteresis loop returns to the same microscopic domain configuration^22^,^23^,^24^.

For the realization of the conceptual DW-based nanodevices, whose performance relies on the precise manipulation of DWs, a deeper understanding of DW dynamics under the influence of disorder is required. A conceptual one-dimensional energy landscape has been often used to describe DW motion in disordered system^12^,^21^,^25^,^26^ because DW motion is determined by the interplay between the driving forces and the complex energy landscape of the system. Although the concept of energy landscape is useful in describing DW motion, a theoretical framework for the analysis of the energy landscape is yet not well established in ferroic systems because of complex interactions between DWs and disorder. Recent reports on the spatial and energy distribution of nucleation centers^27^, nanoscale ferroelectric switching dynamics^11^,^28^,^29^,^30^,^31^, and controllability of switching process^32^ and DW motion^19^,^20^,^21^ have advanced the understanding of domain dynamics in ferroic systems. Nevertheless, practical studies on the energy landscape, which can explain the evolution dynamics of complex domain structure and its correlations with defects, have been rare^29^. The notion of energy landscape will provide a much deeper understanding of DW motion in ferroic systems in which driving forces act.

In this paper, we report an intuitive approach to understanding complex domain dynamics in ferroelectric thin film, including domain nucleation and the propagation of DWs, by visualizing spatial energy landscape that correlates the energy landscape with defect-induced local internal fields. In the scheme of energy landscape, domain switching kinetics corresponds to the process of sequentially occupying the lowest available energy states of polarization under an external field. Our work provides new insights into domain dynamics and highlights a new pathway for controlling DW motion in emerging technologies that exploit DWs by using defect-induced internal fields such as flexoelectric fields.

## Results and Discussion

### Domain-shape-preserving deterministic domain wall motion

**Figure 1:**
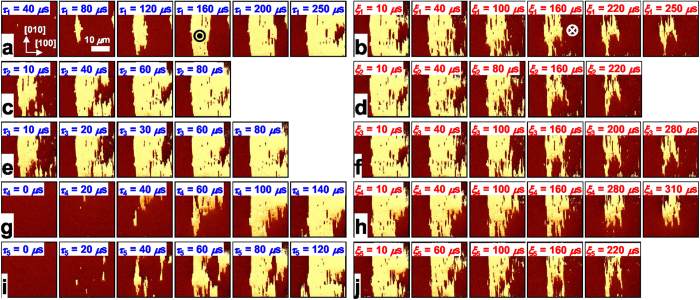
Domain-shape-preserving DW breathing motion. **a-j**, Successive out-of-plane PFM phase (*θ*) images of FE domains taken upon application of each pulse field. Domains expand and shrink in approximately the same patterns under the 1^st^, 2^nd^, 3^rd^, 4^th^, and 5^th^ series of negative (**a**, **c**, **e**, **g**, and **i**) and positive (**b**, **d**, **f**, **h**, and **j**) switching pulses with the respective switching times of *τ*_*i*_ and *ξ*_*i*_ (*i *= 1,2,3,4,5), τ_i_ and ξ_i_ are the cumulative pulse time of each domain-switching with negative and positive switching pulses, respectively, i.e. the sum of the widths of a series of switching pulses (Supplementary Fig. S3a). ⊙ and ⊗ represent the direction of the external upward and downward electric fields induced by negative and positive switching pulses, respectively. The bright and dark regions in the out-of-plane PFM images represent ferroelectric domains with up and down polarization, respectively.

BiFeO_3_ (BFO) is known to have a very strong coupling between ferroelastic strain and ferroelectric polarization^33^. We used epitaxial Pt/BFO/SrRuO_3_ heterostructure grown on a vicinal SrTiO_3_ (STO) (001) substrate with a 2° miscut as a model system (For the detailed information of sample characterization, see Methods and Supplementary Figs. S1 and S2). By using stroboscopic piezoresponse force microscopy (PFM)^34^, we investigated the evoultion of switched domains in the BFO film systematically. Especially, we observed DW breathing, *i.e.*, the repetitive back-and-forth motion of DWs, by applying cyclic series of positive and negative electric pulse fields to the top Pt electrode (see Methods and Supplementary Fig. S3). The PFM phase (*θ*) images in Fig. 1a show the nucleated domains of upward polarization (bright regions) growing anisotropically in a down-poled single domain by applying a series of negative switching pulses. We note that subsequent application of positive pulses causes only the shrinkage of the switched up-domains (Fig. 1b). Repeated measurements (Fig. 1a–j) enable us to trace the positions of DWs during the breathing motion and provide information on the evolution dynamics of complex domain structure.

**Figure 2:**
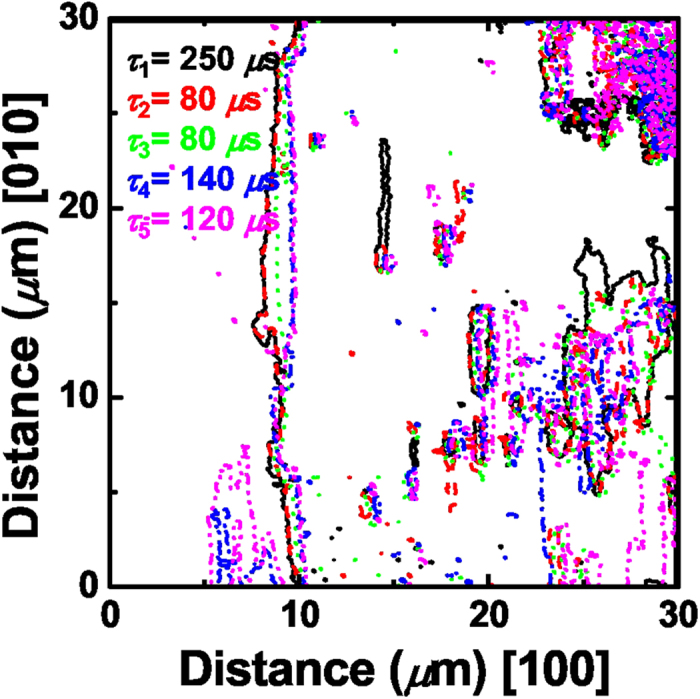
Overlaps of DW positions showing deterministic DW motion. The overlapped positions of DWs obtained from the PFM images in Fig. 1a,e,g,i for *τ*_1_ = 250 μs (solid black lines), *τ*_2_ = 80 μs (dashed red lines), *τ*_3_ = 80 μs (dotted green lines), *τ*_4_ = 140 μs (dash-dotted blue lines), and *τ*_5_ = 120 μs (dash-dot-dotted purple lines). The positions of all the DWs are nearly identical, showing deterministic motion of DWs in the vicinal BFO (001) film.

Importantly, the behavior of DW breathing in the BFO film reveals that DW motion is deteriminstic in large parts. The DW breathing motion shows an asymmetric growth behavior, where DWs at the right side of the switched domain move toward the [100] or [−100] directions, while the left side is strongly pinned, in agreement with the previous report^35^. Although minor differences are found in the detailed shape of domains, the overall evoution of switched domains follows nearly the same pattern during domain expansion and shrinkage. Even for the repetitive nucleation processes, they grow in similar shapes (Fig. 1g,i). Figure 2 displays the overlapped positions of DWs for the largest switched domains, which are determined from the PFM images in Fig. 1a–j. It should be noted that the positions of DWs are practically identical with only small minor variations.

Microscopic return point memory, which measures the reproducibility of microscopic domain structure after hysteresis loop cycling in ferroic systems, is strongly influenced by disorder and has been shown to increase with increasing random-field disorder^22^. The domain-shape-preserving DW breathing motion directly demonstrates a high degree of microscopic return point memory and confirms the strong effect of disorder in the BFO film. The DW motion in the film is largely deterministic and the patterns of domain evolution should be affected by the defects in the film. Namely, the DW motion should be subject to the energy landscape that is pre-determined by the defect-induced built-in internal fields in the film. Therefore, the traces of DWs should correspond approximately to the equipotential lines in the energy landscape of the BFO film, similar to contour lines in a map.

### Energy landscape and built-in internal fields

In order to explore experimentally the features of energy landscape for domain dynamics in ferroelectrics, we first consider the energetics of polarization switching under local effective field, ***E***_*eff*_(***r**, t*).
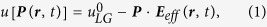

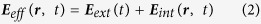
where *u*[***P***(***r***, *t*)] is the free-energy density of polarization ***P***(***r***) and the term 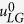 can be expressed as the Landau-Ginzburg-Devonshire form of the Gibbs free-energy density at zero external field. ***E***_*ext*_ and ***E***_*int*_(***r***, *t*) are external and local internal fields, respectively. The origin of the internal field includes depolarization field, ferroelastic strain, and other fields coming from defects *etc*. As for the effect of defects, Nattermann *et al.* introduced random-field and random-bond type interactions between DWs and random disorders to treat DW pinning in disordered systems^7^. The random-field defects break local symmetry by causing built-in internal fields and give rise to different double-well potential energies for equivalent ferroic states. On the other hand, the random-bond defects do not break local symmetry and the potential energy should be the same for equivalent ferroic states.

**Figure 3:**
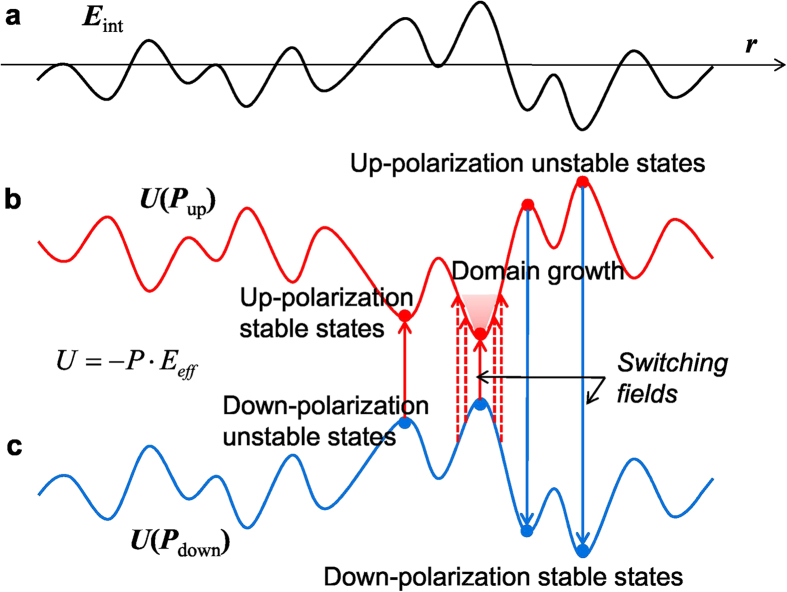
Schematic energetics of polarization in built-in local internal fields. The local energy of polarization in builit-in local internal fields depends on the initial poling states of the polarization. **a**, A presumed one-dimensional profile of the internal field. **b,c** The potential energy profiles of *U*(*P*_up_) (**b**) and *U*(*P*_down_) (**c**) are the local energies for up-polarization and down-polarization poling states, respectively. The external switching field shifts the overall levels of the energy profiles and induces instabilty of the intial states, causing transition from the initial (up/down)-polarization state to (down/up)-polarization state. Nucleation occurs at energetically the most unstable site(s) by the transition of the polarization states, as indicated with the up-/down-arrows. Additional application of the swiching fields causes succesive transitons of polarization states along the paths of the lowest energy states (red dotted-lines), driving the growth of nucleated domain (shadow region).

If we assume that a ferroelectric system is initially poled to have a uniform polarization by applying a strong external field, the local energy of polarization determined by equation (1) should reflect built-in local internal fields. Figure 3a shows a presumed 1-dimensional profile of the internal fields. Then, the profiles of potential energy depends on the initial poling states of polarization, as shown in Fig. 3b,c. The energy profile for up-polarization (Fig. 3b) has the reversed shape of the builit-in internal field, while that for down-polarization poling state (Fig. 3c) resembles the profile of the internal field. Each local energy state corresponds to one of the energy minima of double-well potential that is conventionally used in describing polarization states. The built-in internal fields cause the local double-well potential to become asymmetric. It should be noted that, for polarization switching to occur by applying an external field, an energy barrier must be overcome. The local energy barrier is determined in principle by the energy difference between zero-polarization and ±*P* states in equation (1). An external switching field would induce instabilty of the intial states by shifting the overall energy levels of polarization in the energy landscape. In case of the local effective field being strong enough for the nucleation of opposite domain, a transition from the initial down- to up-polarization state can be initiated at energetically unstable sites by overcoming the energy barrier, as indicated with red up-arrows in Fig. 3b,c. The transition probability would increase with increasing the strength of external field. The nucleation of down-polarization domains in the initial up-polarization state occurs in a similar way, as indicated with blue down-arrows. After the nucleaton process, switched domains continuously grow into nearby regions by occupying the lowest available energy states in the energy landscape. If we use an analogy between the energy landscape for polarization and the geometrical landscape of a lake to explain the growth of domains, the schematic energy landscape for up-polarization state in Fig. 3b corresponds to the geometric landscape in which water has been completely drained away. Then, the expansion of water-filled and exposed (i.e., absence of water) regions would correspond to the growth of up- and down-polarization domains, respectively, upon the application of switching external fields. Hence, the domain switching kinetics of ferroelectric films corresponds to the process of sequentially occupying the lowest available energy states in the energy landscape under an external field, which is dependent on the spatial distribution of the stable/unstable energy states for different polarization.

It should be mentioned that domain growth may influence local internal fields through the interaction between the polarization of adjecent sites^3^,^7^,^8^. The interaction may modify the local energy state of polarization near DWs without affecting signifcantly the overall profile of *U*[***P***(***r***)]. When the interaction is strong enough to overcome the effect of defect-induced internal fields, a stochastic (or non-deterministic) DW motion should occur. Thus, nanoscale study of domain dynamics can provide information on defect-induced internal fields as well as the dominant defect-type governing the domain dynamics.

### Energy landscape and domain switching kinetics

**Figure 4:**
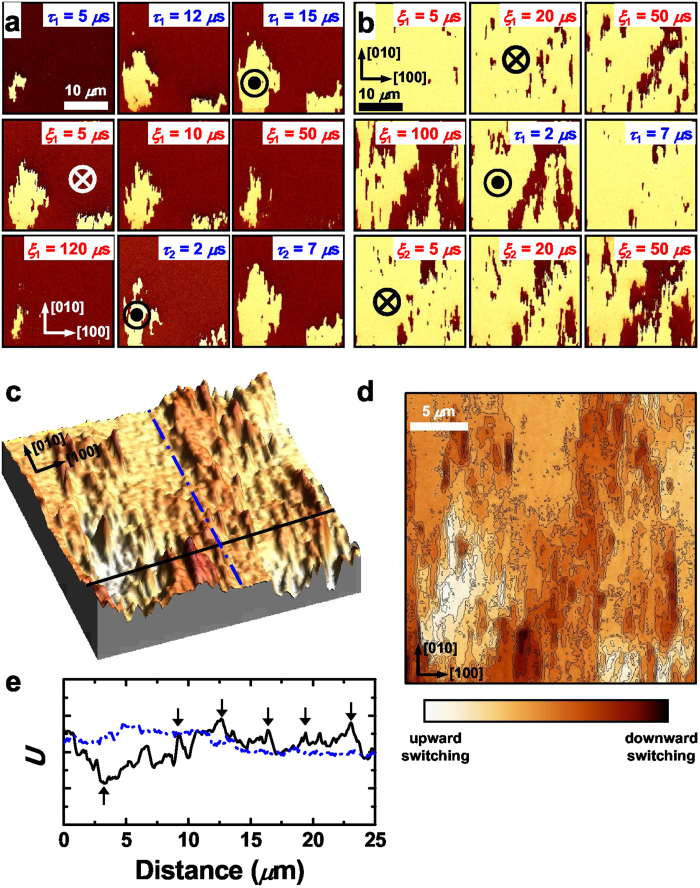
Spatial mapping of energy landscape and the cross-sectional profiles. **a,b** Successive out-of-plane PFM phase (*θ*) images during the breathing motion of DWs for the switched domains from the two different initial single-domain with down- (**a**) and up-polarization (**b**) states. ⊙ and ⊗ indicate the directions of the upward and downward electric fields induced by the negative and positive switching voltages, respectively. The bright and dark regions represent ferroelectric domains with up and down polarization, respectively. *τ*_1_ and *τ*_2_ (*ξ*_1_ and *ξ*_2_) are the cumulative switching times under negative (positive) switching pulses. **c**, The spatial energy landscape (*U*) obtained by superimposing total piezoresponse (*R*cos*θ*) images. **d**, The corresponding contour plot showing equipotential lines in the energy landscape. **e**, Cross-sectional energy profiles along the [100] (solid black line) and [010] (dash-dotted blue line) directions shown in the spatial energy landscape in **c**. The upward and downward arrows represent the preferential nucleation sites of ferroelectric domains with up and down polarization, respectively.

To gain further insights into the details of the deterministic DW motion, we investigate DW breathing for two different polarization states in the out-of-plane direction, as shown in Fig. 4a,b (Supplementary Fig. S4). The domain evoluton of the switched domains also reveals the domain-shape-preserving deterministic motion of DWs. We note that the distribution of nucleation sites are quite different for the different intial polarization states^32^,^34^. This indicates that local polarization reversal symmetry is broken considerably by defect-induced built-in internal fields which lower local nucleation energy. Thus, in a plane-capacitor experiment, random-field type defects, probably formed at the interfaces^31^,^32^, are expected to play a dominant role in domain switching^27^, leading to the deterministic DW motion as well as inhomogeneous deterministic nucleation^11^,^32^,^34^.

The energy landscape of the BFO film can be mapped by superimposing the total piezoresponse (*R*cos*θ*) images acquired during the breathing motion, and then plotting the total intensity of each pixel in spatial coordinates^36^ (see Methods and Supplementary Fig. S5). The deduced energy landscape is shown in Fig. 4c and its contour plot (Fig. 4d) shows the equipotential lines in the energy landscape. It should be mentioned that there exist uncertainties in the energy landscape because DW motion is not perfectly deterministic and depends on the spatial distribution of built-in internal fields. The regions of middle contrast in Fig. 4d have the maximum uncertainty in the positions of DWs for different switching events and a stochastic behavior may appear. On the other hand, in the regions of extreme contrast, DW positions show the minimum variation. The spatially-resolved energy landscape provides the basis for an intuitive understanding of complex domain dynamics.

The nucleation and subsequent growth behavior of domains are determined by the morphology of the energy landscape. Figure 4e shows the cross-sectional profiles of the spatial energy landscape along the [100] (solid black line) and [010] (dash-dotted blue line) directions. The nucleation of up-polarization domain occurs at the local minimum of the energy landscape (marked by upward arrow). The asymmetric shape of the energy landscape profile along the [100] direction well explains the directional growth of up-polarization domains along the direction^35^. The right side of the nucleation site exhibits slow variation in energy and low-energy states are easily occupied by polarization switching, resulting in easy DW propagation toward the [100] direction. On the other hand, a much larger variation of energy at the left side of the nucleation site hinders domain growth to the [−100] direction. For the down-polarization domains, the nucleation sites (downward arrows) are widely distributed within a narrow energy range, which causes a multiple nucleation at the initial stage of switching. The slow variations of energy at the left sides of nucleation sites result in the preferred DW propagation along the [−100] direction, *i.e*., easy occupation of down-polarization states. As for the DW propagation along the [010]/[0–10] direction, the cross-sectional profile of the energy landscape (dash-dotted blue line) is relatively flat for both up- and down-polarization domain switching, which allows fast domain growth to the direction. Thus, the morphology of energy landscape well explains the asymmetric DW propagation and anisotropic domain growth in the film.

### Field-driven domain wall dynamics in the energy landscape

DWs in the energy landscape are interfaces between the occupied and unoccupied energy states of up-/down-polarization. The nontrivial morphology of the energy landscape in Fig. 4c hints at the nature of weak-field DW creep. In a weak field, DW front would propagate along a complex path that follows nearby local energy minima in the energy landscape by a thermally assisted process. The behavior is conventionally described as creep motion of DWs. This motion detours high energy barriers, which results in unswitched islands inside the switched domains in Figs 2a–j and 4a,b. On the other hand, an increase in electric field increases the probability of occupying the higher energy states in a short time by polarization switching, which causes the depinning transition or crossover to the flow regime of DW motion^8^,^9^,^10^. Thus, pinning-dominated driven dynamics of DWs in disordered media can also be easily understood from the energy landscape point of view.

**Figure 5:**
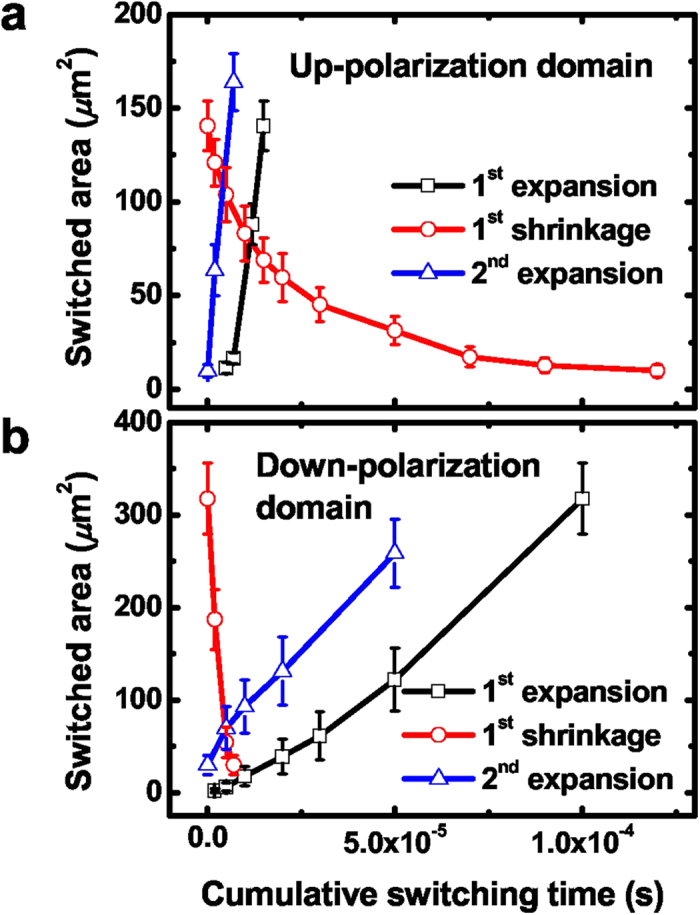
Areas of switched domains during the breathing DW motion. **a,b** The areas of switched domains plotted as a function of cumulative switching time for up- (bright regions) (**a**) and down-polarization (dark regions) (**b**) obtained from the PFM images in Fig. 4a,b. The 2nd expansion after the 1st shrinkage of domains does not include the initial nucleation process while the 1st expansion of domains includes the nucleation process.

We argue that the roughness of energy landscape is also quite relevant to domain growth behaviors. Figure 5a,b shows the areas of switched domains of up- and down-polarization as a function of cumulative switching-pulse time during domain expansion and shrinkage. We first note that the areal speed of domain growth, *v*_*a*_, which is equivalent to the slopes of the time dependence of the switched areas, is quite different during the expansion and shringkage of domains. The difference should result from the built-in internal field which prefer to a particular direction of polarization. The values of *v*_*a*_ are also quite dependent on the polarization direction of switched domains; 13.3 and 3.21 mm^2^ s^−1^ for the up- and down-polarization domains in the initial down- and up-poling states, respectively. For the sideways DW motion, we consider the energy cost 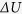 for the nucleation and growth of domains, which can be written approximately as;
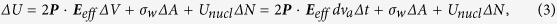
where Δ*V* and Δ*A* are the changes in the volume of switched domains and the area of DWs, respectively, and 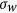 is the average DW energy density. 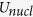 is the average nucleation energy, *d* the thickness of film, and 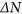 the increase in the number of nuclei. In a rough energy landscape, which is the case of the growh of down-polarization domains (Fig. 4b), multiple nucleation takes place and propagating DWs encounter a large number of high energy barriers. The detouring motion of DWs around the barriers results in large DW areas, requiring additional energy cost for switching in addition to nucleation energy. Therefore, 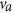 should be small for a given energy supply during the time 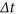 of the switching pulse. Hence, the roughness of the energy landscape can increase the roughnes of DWs and critically affect the growth rate of domains.

Interestingly, the overall switching time varies significantly depending on the preexisting domains. In the second stage of domain expansion (open triangles in Fig. 5a,b), polarization switching is governed only by DW motion due to the preexisting domains. However, if domain switching process includes nucleation (open squares in Fig. 5a,b), 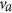 should be much slower according to equation (3) until the nucleation process is completed. Thus, the overall switching time becomes much longer than the domain switching governed only by DW motion. This implies that devices based on DW motion can exhibit much faster operating characteristics.

### Correlation between the energy landscape and disorder

**Figure 6:**
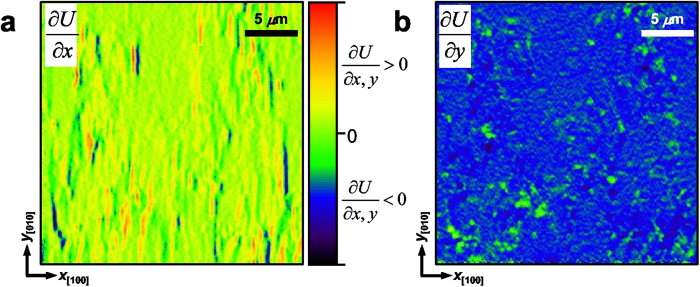
Distribution of local pinning forces in a vicinal BFO(001) film. **a,b** Spatial derivatives of the energy landscape (*U*) with respect to the [100] (**a**) and [010] directions (**b**) of the vicinal BFO (001) film (see Fig. 4c). The spatial derivatives correspond to the pinning forces which impedes the propagation of DWs along [100]/[−100] (**a**) and [010]/[0–10] (**b**) directions. The stripe-like distribution of the pinning force along the [010] direction in **a** shows a correlation with the step-terrace structure of the underlying miscut STO substrate.

Finally, the energy landscape can provide intuitive information on its relationship with disorder in the film. Figure 6 shows the spatial derivatives of the energy landscape, *i.e*., the spatial distribution of pinning forces acting on DW because a steep energy variation hinders DW propagation. For the local pinning forces hindering DW motion toward [100]/[−100] direction, a highly anisotropic stripe-like distribution appears along the step edges of the substrate, parallel to [010] direction (Fig. 6a). On the other hand, no strong pinning force is found for the DW motion toward the [010]/[0–10] direction (Fig. 6b), in consistent with the fast domain expansion along the [010]/[0–10] direction. The anisotropic distribution of the pinning foreces in the vicinal BFO film may be associated with the symmetry breaking induced by the vicinal substrate, because the step-terrace structure of the vicinal STO substrate induces anisotropic strains in the film^35^,^37^,^38^,^39^.

If we consider the asymmetric energy gradients around the nucleation sites in the energy landscape (Fig. 4e), the stripe-like distribution of the local pinnig forces appears to be related to flexoelectric effect. In vicinal BFO films, the anisotropically strained state at the step-edge regions would be relaxed inhomogeneously with the formation of defects such as misfit dislocations^39^,^40^ as the film thickness increases. The asymmetric and anisotropic strain relaxation may give rise to flexoelectric effect in the vicinal BFO film, resulting in the stripe-like distribution of builit-in local field and DW pinning forces (Fig. 6a). Our results are consistent with the recent report that flexoelectric field is weak in the [010] direction compared with that in the [100] direction due to the absence of appreciable strain gradient along the [010] direction for the uniaxially strained vicinal BFO film^38^. This implies that deterministic control of DWs for device applications is possible by engineering the energy landscape using defect-induced internal fields such as flexoelectric field.

## Conclusion

We have investigated the repeated back-and-forth breathing motion of DWs to trace the domain evolution and DW propagation in a vicinal BFO film. Significantly, we demonstrate that the DW motion in the film is largely deterministic, preserving the shape of switched domains. This directly confirms a high degree of microscopic return point memory in the vicinal BFO films. By considering the interaction between polarization and the built-in local internal fields induced by random-field type defects, we define the energy landscape of the vicinal BFO film. In the scheme of the energy landscape, domain switching kinetics corresponds to the process of occupying the lowest available energy states by polarization under an external field. The complex domain dynamics in the film, including domain nucleation and asymmetric/anisotropic growth of domains can be explained by the morphological characteristics of the energy landscape. A stripe-like distribution of the pinnng force for the DW motion toward [100]/[−100] direction, which seems to reveal directly the effect of the interfacial step-terrace structure of the vicinal substrate, was discussed in conjuction with flexoelectric effect. Our approach provides an intuitive understanding of ferroelectric DW dynamics and a pathway to engineering the energy landscape for the novel design of ferroelectric DW devices by controlling defect-induced internal fields such as flexoelectric field.

## Methods

Epitaxial heterostructure film of Pt (40 nm)/BFO (200 nm)/SrRuO_3_ (100 nm) was grown on a vicinal STO (001) substrate with a 2° miscut via off-axis radio frequency magnetron sputtering^41^. The Pt top electrodes, of which area varied from 10 × 10 to 200 × 200 μm^2^, were patterned through photolithography and sputtering. The epitaxial nature of the BFO film was confirmed by using high-resolution x-ray diffraction (AXS D8, Bruker). The preferential monoclinic distortion of BFO lattices toward the downhill miscut direction (i.e., [100]) was also confirmed by reciprocal space mapping (Supplementary Fig. S1). Ferroelectricity of the vicinal BFO (001) film was also confirmed by ferroelectric hysteresis loop measurements (aixACCT TF Analyzer 2000) (Supplementary Fig. S2). Built-in imprint voltage of −0.75 V was revealed in the negative voltage shift of the square-type hysteresis loop.

To visualize the breathing DW motion in a BFO (001) capacitor under the application of an external electric field, a modified stroboscopic PFM configuration was used^34^. For the PFM measurement, we designed a series of electric pulse trains that consist of a poling and switching pulse fields. After applying the initial positive poling pulse, an alternate series of negative and positive switching pulses with constant amplitude *V*_ext_ and a width *t* were applied to the Pt top electrode of BFO capacitors (Supplementary Fig. S3a). The cumulative pulse time (τ_i_ and ξ_i_) for each domain-switching state is the sum of the widths of a series of negative or positiveswitching pulses. We monitored the evolution of ferroelectric domains step by step after the application of each switching pulse by using stroboscopic PFM technique^34^,^42^. Out-of-plane PFM images were taken for the amplitude (*R*), phase (*θ*), and total piezoresponse (*R*cos*θ*) signals under an *ac* voltage of 0.3 V_rms_ at a frequency of 19.1 kHz. The *V*_ext_ values used for polarization reversal were −4.5 and 3.0 V for negative and positive switching pulses, respectively, which were close to the coercive voltages in the ferroelectric hysteresis loop. Additional details of the PFM experiment, including the *R* images that clearly show DWs, can be found in Supplementary Fig. S3b,c.

The successive application of pulse field causes the occupation of the lowest available states in order, and thus, the traces of the DWs obtained from the out-of-plane PFM images correspond to equipotential lines in the spatial energy landscape. By adding up the Rcos*θ* (i.e., piezoresponses with a voltage unit) signals of the PFM images acquired during breathing DW motion pixel by pixel, we were able to map the spatial energy landscape (Supplementary Fig. S5). In detail, the intensity of the Rcos*θ* images was accumulated with the same weight, because each image was obtained after applying almost identical switching pulses in terms of the amplitude and the pulse width. The superimposed Rcos*θ* image was presented spatially and its contour plot based on the accumulated intensity using the WSxM software^36^. In addition, we would like to note that the absolute intensity value in each pixel cannot be solely used to describe the observed domain switching behaviors. Rather, relative intensity differences between adjacent pixels provide the basis for qualitative explanation of domain nucleation and subsequent sideways domain wall motion.

## Additional Information

**How to cite this article**: Kim, T.H. *et al.* Energy landscape scheme for an intuitive understanding of complex domain dynamics in ferroelectric thin films. *Sci. Rep.* **5**, 11625; doi: 10.1038/srep11625 (2015).

## Supplementary Material

Supplementary Information
