# Supplementary material for: Energy landscape scheme for an intuitive understanding of complex domain dynamics in ferroelectric thin films
Source: Sci Rep. 2015 Jul 1;5:11625. doi: 10.1038/srep11625 (PMC4486935; doi:10.1038/srep11625)
Supplement: Supplementary Information [file srep11625-s1.pdf]

Supplementary Information

Energy landscape scheme for an intuitive understanding of complex domain dynamics in ferroelectric thin films

Tae Heon Kim[1,2], Jong-Gul. Yoon[3,*], Seung Hyub Baek[4], Woong-Kyu Park[2], Sang Mo Yang[1,2], Seung Yup Jang[2], Taeyoon Min[2], Jin-Seok Chung[5], Chang-Beom Eom[4], and Tae Won Noh[1,2]

[1]*Center for Correlated Electron Systems, Institute for Basic Science (IBS), Seoul 151-747, Republic of Korea*

[2]*Department of Physics & Astronomy, Seoul National University, Seoul 151-747, Republic of Korea*

[3]*Department of Physics, University of Suwon, Hwaseong, Gyeonggi-do 445-743, Republic of Korea*

[4]*Department of Materials Science and Engineering, University of Wisconsin-Madison, Madison, WI 53706, USA*

[5]*Department of Physics, Soongsil University, Seoul 156-743, Republic of Korea*

* Correspondence and requests for materials should be addressed to J.-G.Y (jgyoon@suwon.ac.kr) or T.W.N (twnoh@snu.ac.kr)

I. Monoclinic distortion of $BiFeO_3$ (BFO) film grown on a vicinal $SrTiO_3$ (STO) (001) substrate.

We confirmed the preferential monoclinic distortion of BFO lattices toward the downhill miscut direction (*i.e.*, [100]), as structural information is essential for the understanding of the energy landscape. The crystallographic structure and the ferroelastic domain configuration in the BFO film with respect to the substrate's miscut direction were determined by performing reciprocal space mappings (RSMs) around the {103} STO Bragg peaks. Figure S1 shows the RSMs in the vicinity of the (103) and (013) STO Bragg peaks. In the (103) RSM [Fig. S1(a)], we observed a single BFO Bragg peak, whereas the BFO peak in the (013) RSM [Fig. S1(b)] was split along the L-direction. The L-scan profiles of the BFO Bragg peaks in Fig. S1(c) clearly showed peak splitting only for the (013) and (0-13) RSMs. This indicates that the BFO unit cells were structurally twinned in only the [010] direction, as shown in Fig. S1(d). The use of a vicinal substrate breaks the symmetry of domain variants, reducing the four structural variants to two, with monoclinic distortion in the [100] direction in a vicinal BFO film. Thus, the substrate's surface symmetry significantly affected the ferroelectric domain structure of the film.

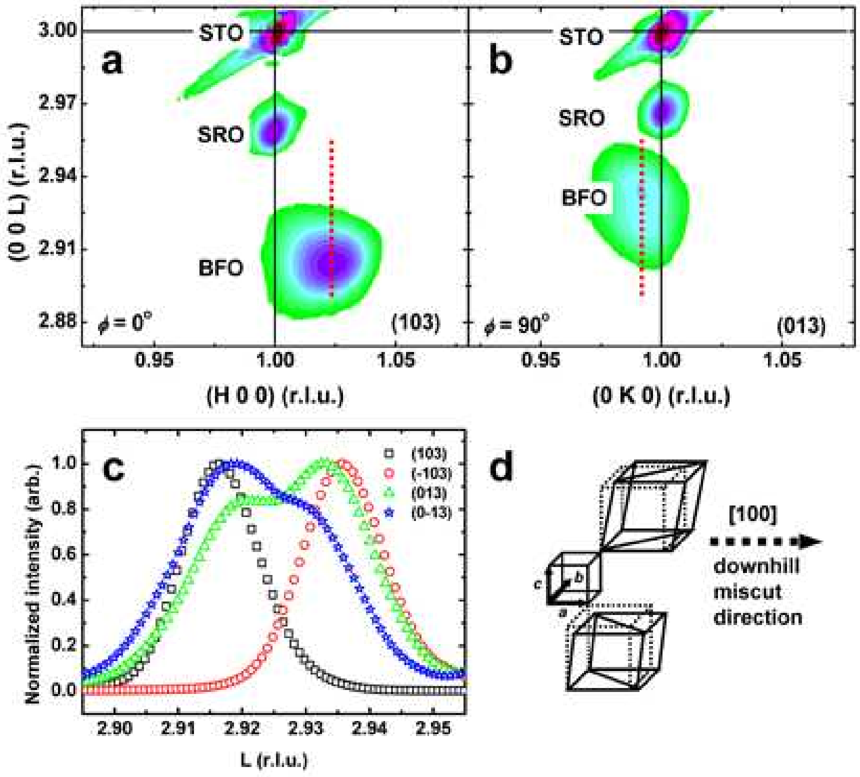

Figure S1 | Structural characterization of a 200-nm-thick BFO film on a vicinal STO (001) substrate with 2° miscut along [100]. RSMs around STO. (a) (103) (ϕ = 0°) and (b) (013) (ϕ = 90°) Bragg peaks in a vicinal BFO (001) film. (c) *L*-scan profiles on the red dotted lines in (a) and (b) around the BFO Bragg peaks in the {103} RSMs. (d) Schematic diagram of a symmetry-broken monoclinic structure in the vicinal BFO (001) film. The *a*-, *b*-, and *c*-axes of the BFO unit cell correspond to the [100], [010], and [001] directions of the vicinal STO substrate, respectively.

II. Ferroelectric polarization (*P*)–voltage (*V*) hysteresis loop of a vicinal BFO film.

We measured *P-V* hysteresis loop for the epitaxial BFO film grown on a vicinal STO substrate at room temperature by applying a triangular wave with the the frequency of 19.1 kHz. The hysteresis loop in Fig. 2S shows remnant polarization value of 75.6 μC/cm^2.

Coercive voltages, V_C^+/V_C^-, were +3.45/-4.71 V, showing an imprint of about -0.63 V. The electrical pulse voltages for domain switching were determined near the coercive voltages.

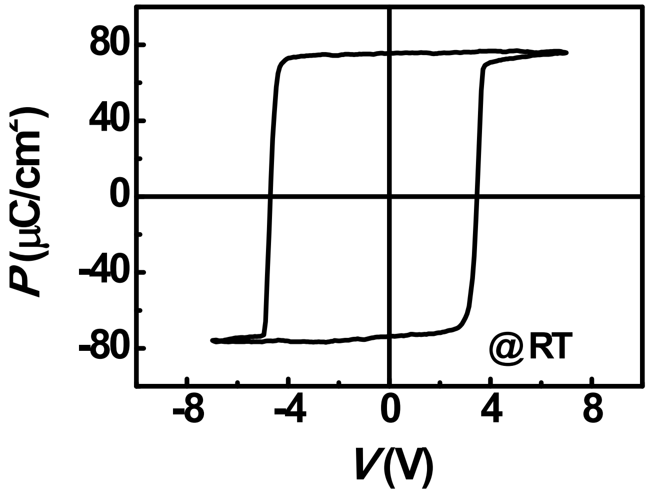

Figure S2 | A ferroelectric *P-V* hysteresis loop of a vicinal BFO (001) film. The loop was measured under a triangular wave with the pulse frequency of 19.1 kHz at room temperature.

III. Step-by-step monitoring of ferroelectric domain evolution in a vicinal BFO film.

To monitor the ferroelectric domain evolution step by step, each switching pulse was applied repetitively, and, out-of-plane piezoresponse force microscopy (PFM) images for the amplitude (R), phase (θ), and total piezoresponse ($R\cos\theta$) signals were captured after each switching pulse. It should be noted that a high poling pulse voltage was applied to make a single domain before switching pulses were applied. The magnitude of pulse voltage, V_{ext}, used for polarization reversal (-4.5 and 3.0 V for negative and positive switching pulses, respectively) was close to the coercive voltages in the ferroelectric hysteresis loop. The corresponding cumulative pulse time for each domain-switching (τ_1 and ξ_1 for the negative and positive switching pulses, respectively) was obtained by the sum of the widths of a series

of negative (i.e., $\tau_1 = \alpha_1 + \alpha_2 + \cdots$) or positive (i.e., $\xi_1 = \beta_1 + \beta_2 + \cdots$) switching pulses in Fig. S3(a). Although we observed the ferroelectric domain evolution using a stroboscopic technique, the switched domain state was preserved before and after the PFM image acquisition without any polarization relaxation or back-switching. This indicates that the PFM images of the breathing motion of the domain walls (DWs) are snapshots of the dynamic switching behavior in response to the external electric field.

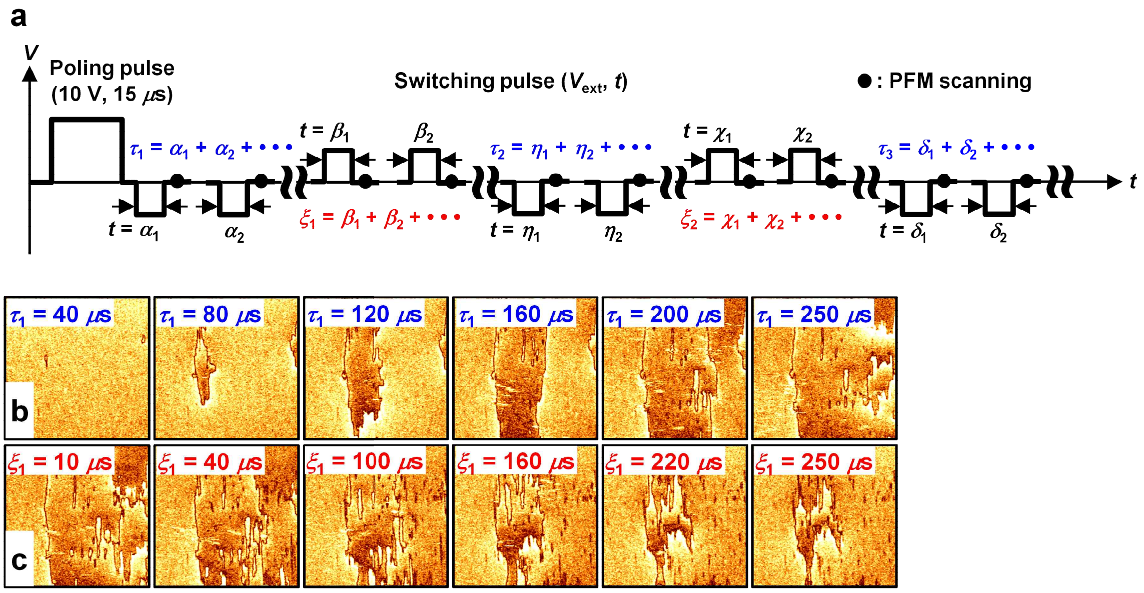

Figure S3 | Electric switching pulse trains for the PFM measurements and the out-of-plane PFM amplitude (*R*) images taken during DW breathing. (a) After the initial poling pulse field, alternative series of switching pulses are applied. (b) and (c) are *R* images of the corresponding phase (θ) images of Fig. 1(a) and (c), respectively. DWs are clearly shown with almost zero amplitude during domain expanding and shrinking.

IV. Polarization dependence of anisotropic DW breathing motion in a vicinal BFO film.

The PFM images for R and $R\cos\theta$ signals for different polarizations were taken to map the spatial energy landscape, as shown in Figure S4. The PFM R signals show exact positions of DWs and the traces of DWs can be determined from the images. If the DW configuration is not exactly vertical to the surface of the film, the positions of DWs is determined at the zero values of $R\cos\theta$.

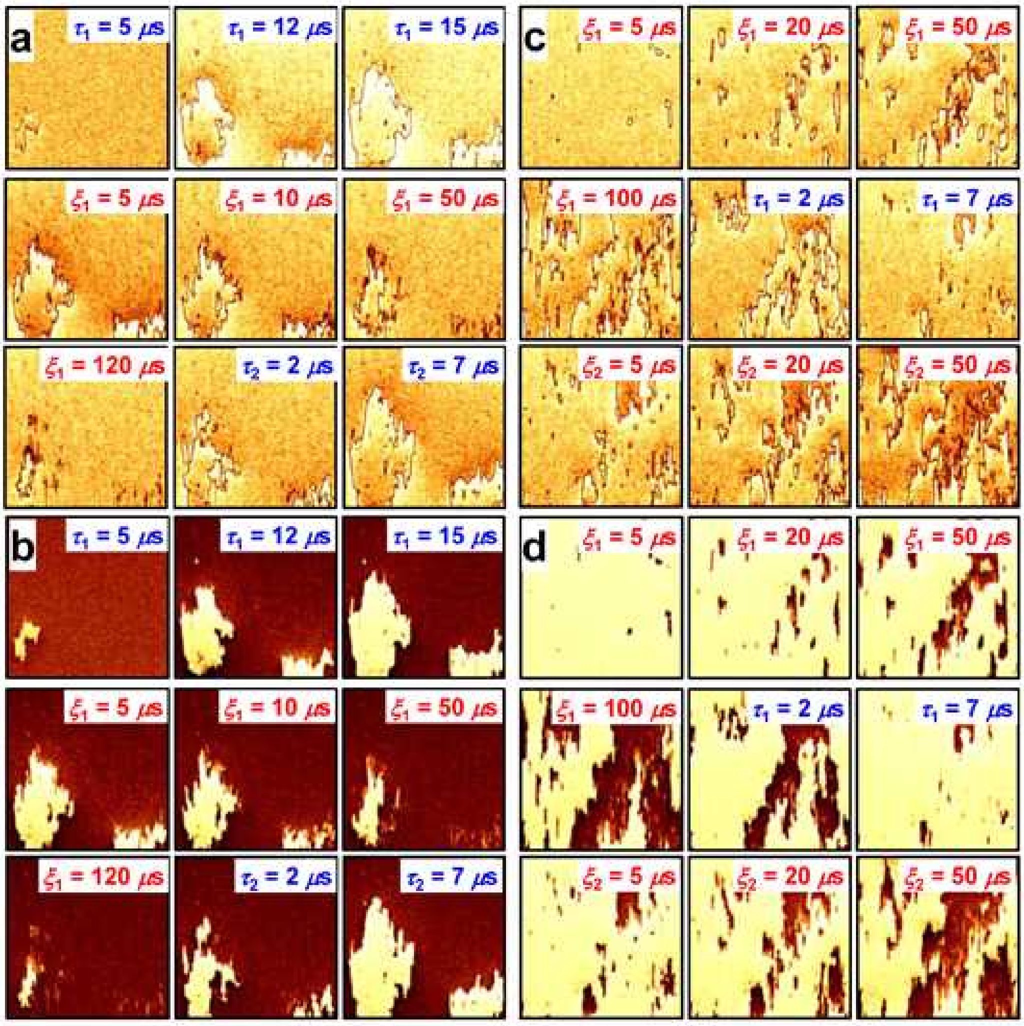

Figure S4 | PFM *R* and *R*cos*θ* images for different polarization states during DW breathing motion in a vicinal BFO film. PFM R [(a) and (c)] and $R\cos\theta$ [(b)

and (d)] images corresponding to the out-of-plane θ images in Fig. 4(a) and (b). As displayed in (a) and (c), DWs are observed while domain expanding and shrinking. In the $R\cos\theta$ images of (b) and (d), propagating domains with up (bright color) and down (dark color) polarization are evident consistently with the θ images of Fig. 4(a) and (b). This indicates that the nucleated domains are fully penetrated across a BFO capacitor in the out-of-plane direction. Thus, the breathing DW motion mainly occurs via sideways movements of ferroelectric DWs.

V. Direct mapping of spatial energy landscape in a vicinal BFO film

The evolution of ferroelectric domains was monitored for both negative and positive switching pulses. The out-of-plane piezoresponse (R, θ, and $R\cos\theta$) images show that nucleated domains are completely extended across the BFO capacitor by forward growth in out-of-plane direction, and thereafter the ferroelectric switching occurs via sideways DW motion (Fig. 1 and 4). It should be noted that the traces of the DWs in each $R\cos\theta$ image, which includes both R and θ signals, can be considered as equipotential lines in the spatial energy landscape for DW motion. By overlapping the obtained out-of-plane $R\cos\theta$ images pixel-by-pixel and plotting the added-up intensity, we mapped the energy landscape in a real space, as shown in Fig. S5.

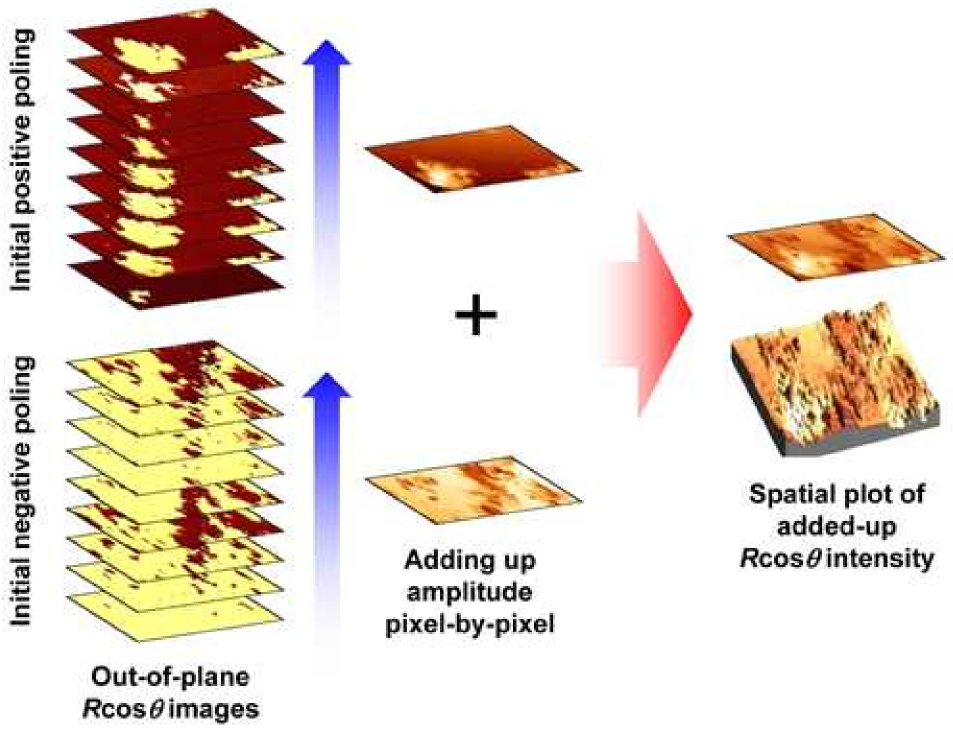

Figure S5 | Out-of-plane PFM imaging and spatial mapping of energy landscape. The total intensity plot of the PFM signals, R$\cos\theta$, which were added pixel-by-pixel, for positive and negative polarization domains results in the spatial energy landscape,
